# Fault detection of high-speed train wheelset bearings based on improved auxiliary classifier generative adversarial networks and VAE

**DOI:** 10.1371/journal.pone.0335368

**Published:** 2025-10-28

**Authors:** Jiandong Qiu, Jiaxuan Liu, Minan Tang, Dingwang Zhang, Meng Li

**Affiliations:** 1 School of Mechanical Engineering, Lanzhou Jiaotong University, Lanzhou, China; 2 School of Automation and Electrical Engineering, Lanzhou Jiaotong University, Lanzhou, China; The Hong Kong Polytechnic University, CHINA

## Abstract

Fault detection in high-speed train wheelset bearings is paramount for ensuring operational safety. However, the scarcity of fault samples limits the accuracy of traditional detection methods. To address this challenge, this paper proposes a supervised generative model that integrates an Improved Auxiliary Classifier Generative Adversarial Network (IACGAN) with a Variational Auto-Encoder (VAE). Firstly, the method employs the VAE as the generator, introducing latent variables with prior information to optimize the encoding and generation process; Secondly, an independent classifier network is integrated into the ACGAN framework to enhance compatibility between classification and discriminative capabilities. Concurrently, a loss function incorporating Wasserstein distance and gradient penalty terms is designed to prevent gradient vanishing during training while satisfying Lipschitz constraints, thereby improving model stability. Experiments conducted on the XJTU bearing dataset validate that samples generated by the proposed method demonstrate superior quality assessment at both the data and feature levels compared to several GAN variants. Furthermore, the constructed VAE-IACGAN-CNN detection model achieves an average classification accuracy of 88.04%, representing a maximum improvement of 15.17% over comparative methods. This significantly mitigates accuracy degradation caused by sample imbalance, demonstrating the proposed approach’s efficacy in resolving low fault detection accuracy stemming from imbalanced high-speed train wheelset bearing samples.

## Introduction

During the operation of high-speed trains, wheel-set bearings bear the sprung mass of the entire train and endure the impact between wheels and rails, resulting in their operation under relatively complex and harsh working conditions [1]. Accidents caused by wheel-set bearing faults are usually severe, which may lead to train derailment, hot box failure, axle burning, and other incidents [2]. The main forms of wheelset bearing faults are as follows: (1) Surface defects on the outer ring, inner ring, or rolling elements, such as scratches, indentations, spalling, and pitting; (2) Local defects of the cage, including deformation or cracking [3]. These faults are not only related to the operational efficiency of the train, but also directly associated with driving safety. Once a fault occurs, the resulting safety hazards are significant; in severe cases, it may cause substantial economic losses and casualties [4–6]. According to the statistics on bearing faults in the transmission systems of China’s high-speed EMUs (Electric Multiple Units) over the past five years, released by the China Academy of Railway Sciences (CARS), the proportions of outer ring faults and inner ring faults in bearings are 42% and 28%, respectively. The fault probability of each part of the bearing varies, and the manifestation of bearing faults is closely related to rotational speed and load; thus, bearing faults are generally highly associated with working conditions [7]. Therefore, to ensure the healthy operation of wheelset bearings in high-speed trains, real-time monitoring and diagnosis of mechanical components have become crucial measures to guarantee the safe operation of high-speed trains.

In practical applications, the detection of high-speed train wheelset bearings is dominated by online real-time monitoring, supplemented by offline periodic detection. The core technical approaches can be categorized into four types, as detailed below: (1) Vibration signal monitoring: Accelerometer sensors installed on bearing housings are used to collect vibration signals in real time. Subsequent analyses include manual inspection of the vibration spectrum (e.g., identifying the presence of fault characteristic frequencies), as well as calculation of the root mean square (RMS) value or peak value of vibration. An alarm is triggered when these values exceed the preset threshold. (2) Temperature monitoring: Infrared sensors or thermocouples are employed to monitor the temperature of bearing housings in real time. An alarm is activated once the temperature exceeds the specified threshold. (3) Oil analysis: For wheel-set bearings equipped with lubrication circulation systems (e.g., bogie bearings of some Electric Multiple Units (EMUs)), lubricating oil samples are collected periodically to detect the concentration and size of metal particles in the oil. A sudden surge in particle concentration indicates the presence of wear faults in the bearings. This approach is only applicable to offline periodic maintenance. (4) Acoustic monitoring: Microphones are used to collect the operational noise of bearings, followed by analysis of abnormal frequencies in the noise spectrum (e.g., high-frequency noise induced by faults).

In recent years, machine learning-based fault diagnosis has increasingly established itself as a primary technology for fault detection, primarily consisting of two essential steps: fault feature extraction and fault pattern classification.Common feature extraction methods include wavelet transform (WT), principal component analysis (PCA), short time Fourier transform (STFT), etc. Li et al. [8] introduced an improved ensemble empirical modal decomposition (EEMD) time-frequency analysis method based on the improved adaptive resonance technique (IART) to improve the denoising capability of fault-related impulse signals. Lin et al. [9] proposed a cascaded model based on One-Class Support Vector Machine (OCSVM) and Random Forest (RF), which can address the misjudgment issue of traditional methods caused by insufficient samples. Furthermore, the adaptive strategy of directly inputting high-dimensional features into the RF can effectively process high-dimensional data while maintaining high accuracy. Nonetheless, With the rapid development of sensor measurement technology and the rapid improvement of computer performance, traditional fault diagnosis methods have gradually revealed their limitations. Especially when dealing with complex systems and big data, they are often limited by the difficulty in automatically extracting meaningful features; moreover, when facing high-dimensional data and complex nonlinear patterns, they may be unable to effectively capture and analyze the deep-seated features and interactions in the data. All the aforementioned methods belong to shallow learning methods, which have high requirements for expert empirical knowledge and manual feature extraction. Specifically, although Wavelet Transform (WT) can effectively extract nonlinear time-frequency features, it is necessary to select appropriate basis functions when processing complex vibration signals; although Support Vector Machine (SVM) can efficiently solve high-dimensional nonlinear decision-making problems, its kernel parameters and penalty parameters are difficult to select, and it is easily significantly affected by fault samples; although Ensemble Empirical Mode Decomposition (EEMD) can effectively suppress mode mixing and does not require preset basis functions, the amplitude parameters and the number of ensembles need to be manually set, and the end effect still exists. This is mainly attributed to the following three aspects. First, compared with the bearing vibration signals of ordinary equipment, the signals collected from train wheel-set bearings have nonlinear and non-stationary characteristics, along with stronger noise. Second, the operating conditions of the equipment change constantly during operation, resulting in dynamic, complex, and variable fault features. Third, the feature distribution of data samples usually differs under different operating conditions, which is typically manifested in different load conditions. To address this shift, intelligent fault diagnosis approaches leveraging deep learning have emerged and gained prominence as a significant research focus over the past five years. Characterized by a deep architecture consisting of multilayer nonlinear data processing units and the capability to autonomously extract features from raw data, deep learning has emerged as a highly promising tool for bearing fault diagnosis [10–12]. Various deep learning models, including deep belief networks [13], auto-encoders [14], convolutional neural networks [15], and long short-term memory networks [16], have demonstrated significant success in the domain of fault diagnosis.Peng et al. [17] proposed a deep one-dimensional convolutional neural network based on one-dimensional residual blocks, which can effectively learn high-level abstract features as well as alleviate the problems of training difficulties and performance degradation of deep networks.Yao et al. [18] proposed a concurrent fault identification model for wheel-set axle boxes based on efficient neural networks and attention mechanisms. This model adaptively refines feature maps using the Convolutional Block Attention Module (CBAM), thereby enhancing the model’s generalization ability; in addition, it adopts the Feature Pyramid Network (FPN) to fuse shallow and deep features in the network, which improves the ability to extract features of various sizes. Pan et al. [19] proposed an improved bearing fault diagnosis method based on CNNs and Long Short-Term Memory Recurrent Neural Networks (LSTM-RNNs). Through an end-to-end learning mode, this method can automatically extract features from high-dimensional data without any preprocessing, capture the long-term temporal dependencies of fault signals, and solve the gradient vanishing problem of RNNs. However, the training process of the above deep learning models is highly dependent on rich labeled datasets. These labeled sample-based approaches are known as supervised learning strategies [20–22]. To effectively train the numerous parameters inherent in these strategies, it is essential to have an adequate supply of fault marker samples. However, in actual industrial environments, the sample ratio between fault data and healthy data of train wheelset bearings is extremely imbalanced. The challenges mainly stem from the following three dimensions: (1) The high reliability of high-speed train wheelset bearings leads to an extreme scarcity of natural fault samples. As critical safety components, wheel-set bearings comply with the strictest design, manufacturing, and maintenance standards, resulting in an extremely low probability of functional failure during their rated service life. Consequently, it is difficult to obtain sufficient real full-life-cycle data that covers different fault modes (e.g., inner ring, outer ring, and rolling element spalling) and different degradation degrees, making it hard to construct an adequately comprehensive training set for diagnostic models. (2) The architectural design of existing monitoring systems focuses on safety early warning rather than fault analysis, leading to insufficient data dimension and depth. Currently, mainstream on-board detection systems usually adopt a threshold-triggered mechanism, which only records transient data when parameters exceed the threshold. They lack the capability to collect and store high-frequency, continuous time-series data (such as vibration signals) that contain abundant fault information. The limitations of this data acquisition mode make it difficult to effectively capture and analyze the early evolution characteristics and degradation trends of faults [23]. (3) Industry data barriers and commercial confidentiality restrict data accessibility, as such data is usually under strict internal control. The scarcity of data prevents models from receiving sufficient training, which causes models to suffer from overfitting and further seriously impairs the classification performance of these methods [24–26].

Given that labeled fault samples constitute only a small fraction of the data available in real industrial settings, an increasing number of researchers are focusing their investigations on datasets characterized by a scarcity of labeled samples. The Generative Adversarial Network (GAN), introduced by Goodfellow, serves as a pivotal technique in the realm of unsupervised generative modeling. It is capable of synthesizing additional samples that resemble existing data without the necessity for labeled information, thereby enhancing the diversity and quantity of generated samples [27]. Since then, more and more variants of GAN have also emerged and started to be used in various applications. Lee et al. [28] proposed a fault detection and diagnosis scheme for induction motors based on Deep Neural Networks (DNNs) and Generative Adversarial Networks (GANs). Specifically, the Empirical Mode Decomposition (EMD) of the Hilbert-Huang Transform (HHT) is used to extract sensitive fault features, and a 4-layer DNN is designed to realize motor state classification. Four oversampling methods (SMOTE, ADASYN, GAN, and ADASYN+GAN) are compared to address the data imbalance problem and improve the fault detection accuracy of induction motors. Ma et al. [29] proposed a method called Binomial Adversarial Representation Learning (BARL) for solving the problem of feature extraction and diagnosis of machine faults, which combines an adversarial learning mechanism and a self-encoder to extract the binomial distribution representations of the original monitoring signals by learning the key features that contain information about the health of the machine. Shao et al. [30] designed a novel stacked auto-encoder (NSAE) and optimized it by a particle swarm algorithm for solving the problem that the data sets in the source and target domains of the fault signals have significant distributional differences among different rotating machines. Meng et al. [31] introduced a convolutional block attention module that leverages an enhanced auxiliary classification generative adversarial network, incorporating an attention-based mechanism to address the challenges associated with small sample sizes in the domain of bearing fault diagnosis. Tong et al. [32] proposed ACGAN combined with spectral normalization. Meanwhile, label information is used to impose label constraints on the generated fake data, and a generation module is constructed by integrating transposed convolution layers to generate high-quality labeled fake fault data from Gaussian noise—this effectively solves the small-sample and data imbalance issues in bearing fault diagnosis. Zhang et al. [33] proposed a comprehensive multi-task intelligent bearing fault diagnosis framework based on representation learning, which enhances the bottleneck layer through an advanced denoising auto-encoder that employs a self-attention mechanism. The solution is used to solve the problem of bearing fault detection, classification and unknown fault identification under unbalanced sample conditions by enhancing the representation capability of the bottleneck layer through a modified denoising self-encoder, i.e., a self-attention mechanism.

Despite the considerable success of the aforementioned methods in addressing small sample fault diagnosis challenges, several issues remain that warrant further improvement. (1) The classical ACGAN structure in which the discriminator under-takes both discrimination and classification tasks, i.e., the neural networks of the discriminator and classifier share hyper-parameters and weights, which will have an impact on the quality of the final generated samples in the event of an error in one of them [34]. (2) Since the input to the generator in the classical GAN structure is random Gaussian noise, the generator will tend to generate some specific, high-probability outputs and ignore other possible output patterns. This can result in a diminished diversity within the generated data, manifesting as the phenomenon of mode collapse [35]. (3) The structural loss function employed in the majority of Auxiliary Classifier Generative Adversarial Networks (ACGANs) is formulated based on the Jensen-Shannon (JS) divergence. However, this approach is susceptible to instability and gradient vanishing issues during the training process, primarily due to its reliance on discrete representations [36]. (4) Weight Clipping is used in GAN to satisfy the Lipschitz continuity condition, which leads to reduced model flexibility and limited generalization capabilities [37].

To address the aforementioned issues, this paper presents a data enhancement method that fuses the Auxiliary Classifier Generative Adversarial Network and Variational Auto-Encoder and combines them with convolutional neural network for the fault detection of wheelset bearings in high-speed trains. First, VAE encodes the real data and uses the hidden variables obtained after encoding instead of Gaussian noise as inputs to the generator, which improves the generalization ability of model generation and avoids pattern collapse. Further, the incorporation of an independent neural network as a classifier enhances the compatibility between classification and discrimination tasks, thereby mitigating the impact on the quality of generated samples. In addition, a new loss function is designed based on the Wasserstein distance to avoid gradient vanishing during training [38]. Meanwhile, a gradient penalization strategy is used to limit the weight parameters of the discriminator to satisfy the Lipschitz continuity condition [39]. Finally, the VAE-IACGAN model is integrated with a convolutional neural network (CNN) to facilitate fault classification. The principal contributions of this paper can be articulated as follows:

(1) We proposed a novel data enhancement model based on improved ACGAN with VAE, which is able to expand the fault sample dataset and effectively solve the sample imbalance problem [40].

(2) Improved structure of the classical ACGAN model by adding a new neural network as a classifier, improved compatibility of recognition and classification accuracies, and enhanced generation of high-quality multimodal data.

(3) A novel loss function is formulated utilizing the Wasserstein distance, effectively addressing the issue of gradient vanishing. Additionally, a gradient penalization strategy is employed to constrain the weight parameters of the discriminator [41], thereby enhancing the stability of the training process.

(4) The efficacy of the proposed methodology for fault diagnosis in wheelset bearings of high-speed trains is substantiated through experimental validation.

The rest of the paper is organized as follows. The Theoretical Background section describes the basic principles of GAN, classical ACGAN, VAE and Wasserstein distance. The Improved methodology section details how to improve the ACGAN structure and fuse VAE and IACGAN to build a new generative model. The Experimental validation section gives the experimental validation and analysis results. Finally, the Conclusion section summarizes the whole paper and gives an outlook for future work.

For ease of reference, the main abbreviations and symbols used in this paper are listed in the Table 1.

**Table 1:** Main abbreviations and symbols.

Abbreviations and Symbols	Full name
WT	wavelet transform
PCA	principal component analysis
GAN	generative adversarial network
VAE	variational auto-encoder
STFT	short time Fourier transform
CNN	convolutional neural network
ACGAN	auxiliary classifier GAN
IACGAN	improved ACGAN
G	generator
D	discriminator
C	classifier
JS divergence	Jensen-Shannon divergence
ReLU	rectified linear unit
BF	ball bearing fault
IF	inner race fault
OF	outer race fault
SSIM	structural similarity index
MMD	maximum mean difference
PSNR	peak signal to noise ratio
T-SNE	T-distributed stochastic neighborhood embedding

## Theoretical background

In order to solve the problem of relatively limited training samples in real industrial environments, where Adversarial Generative Network (GAN) and Variational Auto-Encoder (VAE) are used as two powerful generative models that are able to generate more training samples. However, both models have their limitations, so this paper proposes to fuse the two models together with the aim of generating higher quality samples. In this section, the concepts of Adversarial Generative Network (GAN) and Auxiliary Classifier Generative Adversarial Network (ACGAN) as well as Variational Auto-Encoder (VAE) are briefly introduced to lay the groundwork for the methods proposed later.

### Adversarial generative network

**Fig 1:**
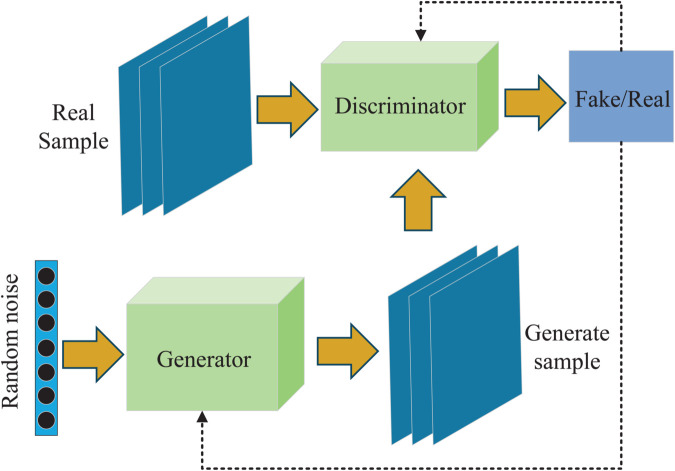
Network structure of GAN. Driving the generator to progressively approximate the real data distribution through adversarial training of the generator and the discriminator.

The GAN is a powerful deep learning model that generates new, similar to real data by pitting two neural net-works against each other [42]. These two networks are the Generator (G) and the Discriminator (D), which each perform different tasks. The goal of the generator (G) is to generate data that is as realistic as possible. The generator receives a random noise vector as input and applies a neural network to transform this vector, ultimately producing novel data samples. The generator tries to trick the discriminator into thinking that the generated data is real. The task of the discriminator (D) is to distinguish whether the data samples are real or generated by the generator. The discriminator inputs both the real data and the outputs generated by the generator, con-ducting a binary classification to ascertain the authenticity of each sample. During training, these two networks work against each other to improve their respective performance through iterative optimization until a dynamic equilibrium, known as the Nash equilibrium, is achieved. The general structure of a GAN is shown in Fig 1, and the loss function is defined as:

$$minmaxV(D,G)=E_{x∼P_{r(x)}}[\log D(x)]+E_{Z∼P_{z(z)}}[\log\left(1-D(G(Z))\right)]$$ (1)

where *P*_*r*_(*x*) is the distribution of the true sample *x*, *P*_*z*(*z*)_ is the distribution of the random Gaussian noise *z*. *D*(*x*) is the probability that the discriminator recognizes the true sample *x* as true, and *D*(*G*(*Z*)) is the probability that the discriminator recognizes the generated sample *G*(*Z*) as true. $E_{x∼P_{r}(x)}(\cdot )$ is the expectation computed under the distribution *P*_*r*_(*x*) of the true sample *x*, and $E_{Z∼P_{z}(z)}(\cdot )$ is the expectation computed under the distribution *P*_*z*_(*z*) of the randomized Gaussian noise *z*.

**Fig 2:**
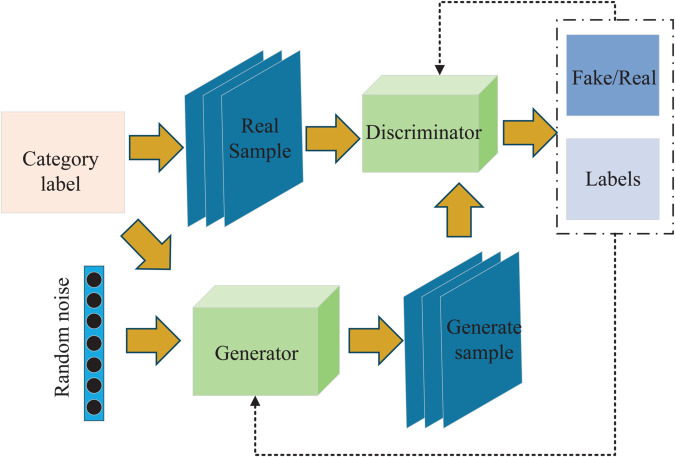
Network structure of ACGAN. ACGAN adds classifier function to the discriminator on the basis of GAN, so that the generator not only generates realistic data, but also learns to generate samples of a specified class, realizing supervised conditional generation.

The ACGAN represents a supervised enhancement of the traditional GAN, as illustrated in Fig 2. The architecture of ACGAN comprises both a generator and a discriminator. In contrast to conventional unsupervised GANs, ACGAN incorporates fault information labels alongside random Gaussian noise in the generator’s input, thereby facilitating the generation of more targeted outputs. The generator is thus able to leverage the auxiliary labeling information to generate higher-quality data, while the discriminator includes an auxiliary network specifically designed to process class information. This dual approach enhances the model’s ability to produce more relevant and informative outputs [43,44]. Thus, the discriminator has the ability to classify and judge truth and falsehood. The loss function of ACGAN consists of two parts, classification loss and discrimination loss, defined as:

$$L_{Source}=E_{x∼P_{r}(x)}[logD(x)]+E_{z∼P_{z}(z)}[log(1-D(G(z,c_{g})))]$$ (2)

$$L_{Class}=E_{x∼P_{r}(x)}[-logP(c=c_{r}|x)]+E_{z∼P_{z}(z)}[-logP(c=c_{g}|G(z,c_{g}))]$$ (3)

where *L*_*Source*_ is a loss function indicating the authenticity of the sample and *L*_*Class*_ is a loss function indicating the classification accuracy of the sample. *P*_*r*(*x*)_ is the distribution of the true sample x and *P*_*z*(*z*)_ is the distribution of the random Gaussian noise *z*. *D*(*x*) is the probability that the discriminator recognizes the true sample *x* as true, and *G*(*z*,*c*_*g*_) is the random Gaussian noise with labels fed in to generate the sample. *c*_*r*_ and *c*_*g*_ are the labels corresponding to the real sample *x* and the generated sample *G*(*z*,*c*_*g*_) respectively. *P*(*c* = *c*_*r*_|*x*) is the probability that the discriminator identifies the real sample *x* with the label *c*_*r*_ and $P(c=c_{g}|G(z,c_{g}))$ is the probability that the discriminator identifies the generated sample *G*(*z*,*c*_*g*_) with the label *c*_*g*_.

During adversarial training, the loss function of the discriminator is $L_{Source}\;+\;L_{Class}$, this is because the discriminator wants to minimize the probability of the real data being judged true, while maximizing the probability of the generated data being judged false and wants to be able to correctly classify both the real data and the generated data. So the discriminator needs to maximize $L_{Source}\;+\;L_{Class}$. The generator loss function is $-L_{Source}\;+\;L_{Class}$. This is because the generator wants the data it generates to trick the discriminator into thinking that the data is real and wants the generated data to be correctly classified by the discriminator. So the generator needs to maximize $-L_{Source}\;+\;L_{Class}$.

### Variational auto-encoder

**Fig 3:**
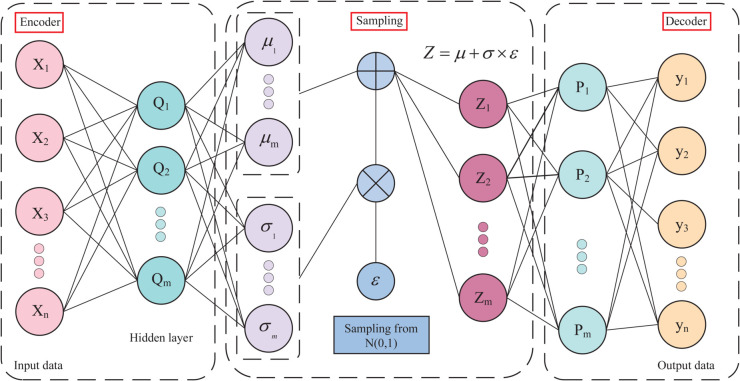
VAE structural model. The VAE learns the latent distribution of the data through the encoder, and the decoder samples and reconstructs the new data from that distribution for controlled generation and implicit feature extraction.

VAE is a deep learning model that combines the concepts of deep learning and probabilistic graphical models. VAE is not only able to learn the representation of data, but also able to generate new instances of data, it has the same structure as a self-encoder, which consists of an encoder, a decoder, and a hidden space, and, VAE adds a sampling layer, which allows it to draw samples from the latent space of the hidden layer, and thus generate new data [45]. The structure of the VAE model is shown in Fig 3.

The encoder part of the VAE is a neural network whose goal is to capture the features of the input data and find a good topology for these features in the potential space. The structure should possess the ability to maintain similarity between input features, meaning that similar input data should be close to each other in the latent space. The encoder maps the input data into a distribution within the latent space, typically characterized by its mean and variance. This distribution encapsulates the statistical properties inherent in the input data. Through a reparameterization technique, the VAE samples from the distribution output by the encoder to generate points in the latent space.

This sampling process introduces randomness, allowing the model to explore the diversity of the potential space. The decoder segment takes the output produced by the encoder as its input and aims to reconstruct the original data. The goal of the decoder is to minimize the difference between its output and the original input data. Throughout the training process, the VAE optimizes the weights and biases of both the encoder and decoder through the application of a backpropagation algorithm. The objective of this process is to minimize the objective function, thereby ensuring that the output generated by the decoder closely approximates the original input data. In this way, the VAE not only learns a valid feature representation of the input data, but is also able to generate new instances of the data that are characteristically consistent with the training data.

Among them, the objective function of VAE is to minimize the distance between the data distribution *P*(*x*) and the reconstructed sample distribution $P(\hat{x})$, and the distance between these two distributions is generally measured using the Kullback-Leibler (KL) divergence:

$$D_{KL}(P(x)\|P(\hat{x}))=\int P(x)\frac{P(x)}{P(\hat{x})}\;dx$$ (4)

Since the unknown nature of the data distribution makes the KL divergence impossible to calculate directly, the proposal distribution *Q*(*x*) (approximate distribution) and the approximate posterior distribution Q(z|x) are introduced. Optimizing the objective function through maximum likelihood yields the log-likelihood function:

$$\log P(x)=D_{KL}\left(Q(z|x)\|P(z|x)\right)+L(x)$$ (5)

Based on the non-negativity property of the KL divergence, it can be derived that logP(x)≥L(x); thus, *L*(*x*) is referred to as the variational lower bound of the likelihood function. After derivation, the variational lower bound can be expressed as:

$$L(x)=-D_{KL}(Q(z|x)\|P(z))+E_{Q(z|x)}(\log P(x|z))$$ (6)

Calculating the second term of the variational lower bound requires sampling from Q(z|x), yet the sampling operation is non-differentiable and cannot enable parameter optimization through backpropagation. For this reason, the reparameterization method is proposed. Reparameterization converts the stochastic samples obtained by sampling from this distribution into deterministic samples; sampling from a simple distribution can reduce the computational complexity of the sampling process. Specifically, by selecting P(ε) from the same probability distribution family, equivalent results to those obtained by sampling from the original distribution can be achieved through performing a number of linear transformations on the samples *ε* sampled from P(ε).

Let P(ε)∼𝒩(0,1), and sample *L* samples $\varepsilon^{i}$ from P(ε); then $z^{i}=\mu \;+\;\varepsilon^{i}\;\times \;\sigma$, which avoids direct sampling. At this point, the estimator of the second term of the variational lower bound can be expressed as follows:

$$E_{Q(z|x)}(\log P(x|z))≃\frac{1}{L}\sum_{i=1}^{L}F\left(\mu +\varepsilon^{i}\times \sigma \right)$$ (7)

where ε∼N(0,1), Typically, setting *L* to 1 is sufficiently precise. That is, the latent variables sampled in each training iteration are randomly generated; when the number of training iterations is sufficient, the sufficiency of sampling can be satisfied to a certain extent. Thus, *L*=1 can meet the training objective of the VAE.

As shown in Fig 3, X denotes the original input data of the model; Q represents the intermediate representation obtained by computing the input data through the encoder, where m stands for the dimension of this hidden layer; Z is the latent variable generated after reconstructing the original data X; *μ* and *σ* denote the mean and standard deviation, respectively, which are characteristic parameters describing the distribution of latent variables; *ε* represents the random noise sampled from the standard normal distribution, and its dimension is the same as that of the latent variable Z; y denotes the reconstructed output data.

VAE digs deeper into the intrinsic structure of the data through its network of encoders, thus grasping the distributional characteristics of the data, i.e., being able to grasp the corresponding noise distribution of various data. In terms of data pre-processing, VAE is able to use the learned noise distribution to create new samples. However, the VAE primarily depends on the mean squared error between the generated samples and the original data during the data generation process. The absence of adversarial learning mechanisms may result in a reduction in the quality of the generated outputs.

### Wasserstein distance

In the original GAN structure, Jensen-Shannon (JS) divergence is typically utilized to assess the distributional distance between generated samples and real samples. However, the discrete characteristics inherent in JS divergence may result in challenges such as gradient vanishing and mode collapse during the training process.

Consequently, Arjovsky et al. [46] introduced the Wasserstein Generative Adversarial Network (WGAN), in which the loss function is formulated based on the Wasserstein distance rather than the traditional JS divergence. One of the advantages of the Wasserstein distance is its greater smoothness, which enables the provision of meaningful gradients even in scenarios where the two distributions do not overlap in high-dimensional space. In traditional GANs, JS divergence can cause the generator to end up generating samples of only a few patterns, ignoring the rest of the data distribution. The Wasserstein distance encourages the generator to generate samples of the entire data distribution, thus avoiding pattern collapse. In WGAN, the discriminator no longer simply determines whether a sample is real or generated, but evaluates the potential value of the sample distribution. This allows the discriminator to focus more on the differences in the distributions during the optimization process, rather than simply classifying them. The formula for the Wasserstein distance is as follows:

$$W(P_{x},P_{y})=\underset{\gamma ∼\Pi (P_{x},P_{y})}{\inf}\left[{𝔼}_{(x,y)∼\gamma }\left(\|x-y\|\right)\right]$$ (8)

where $W(P_{x},P_{y})$ is the Wasserstein distance between the distribution *P*_*x*_ of the true sample *x* and the distribution *P*_*y*_ of the generating sample *y*; $\inf_{\gamma ∼\Pi (P_{x},P_{y})}(\;\cdot \;)$ denotes the minimization of the expectation; where $\Pi (P_{x},P_{y})$ is the set of joint probability distributions *γ* of *P*_*x*_ and *P*_*y*_; ${𝔼}_{(x,y)∼\gamma }\left(\|x\;-\;y\|\right)$ denotes the expected distance between *x* and *y* under the *γ* distribution. In practice, in order to ensure that normal gradient backpropagation can be performed using the Wasserstein distance, the change in its gradient needs to satisfy the K-Lipschitz condition with the following expression:

$$W(P_{x},P_{y})=\underset{∥D{∥}_{Lip\leq 1}}{\sup}\{E_{x∼P_{x}}[D(x)]-E_{y∼P_{y}}[D(y)]\}$$ (9)

where sup(·) denotes taking the maximum value, and ${\left\|D\right\|}_{Lip\leq 1}$ denotes the 1-Lipschitz constraints imposed on the discriminator D to enable it to satisfy the constraints required by the Lipschitz continuity condition.

## Improved methodology

This chapter proposes an improvement of the ACGAN structure and combines it with VAE to form a new model for sample expansion, while designing a new loss function based on the Wasserstein distance and gradient penalty, and finally fault identification by convolutional neural network.

**Fig 4:**
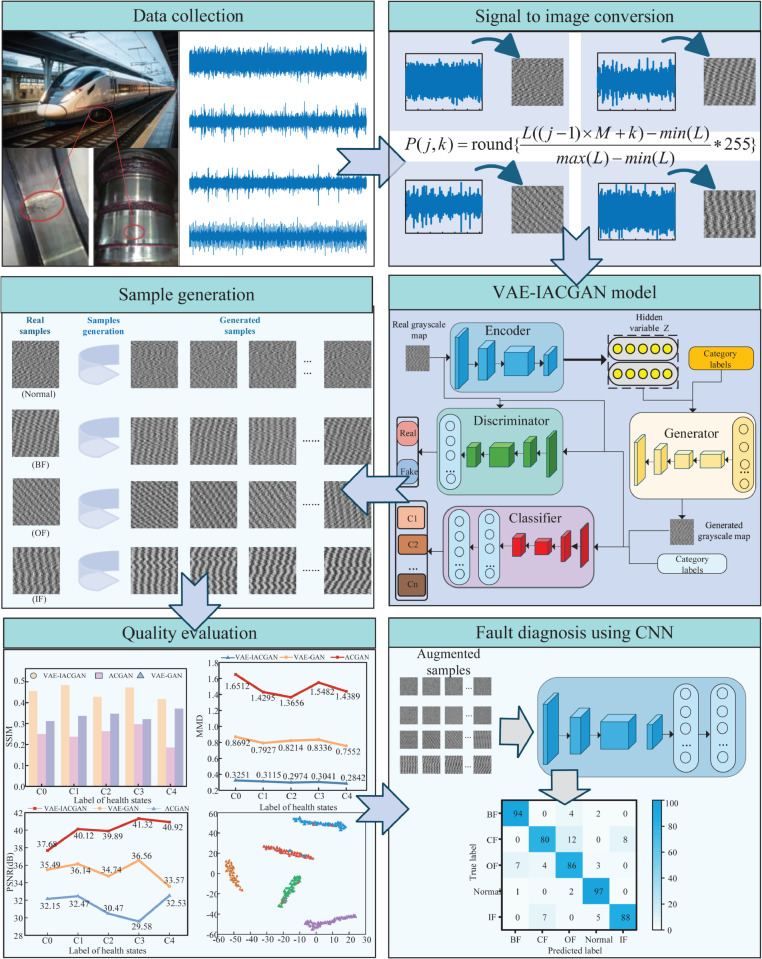
Overall framework of the proposed methodology. The procedure involves three primary stages: initial data preprocessing, followed by sample augmentation via a VAE-IACGAN model accompanied by quality assessment of the generated samples, and concludes with fault classification using CNN.

The overall framework of the method proposed in this paper is shown in Fig 4.

### Improvement of the ACGAN structure

**Fig 5:**
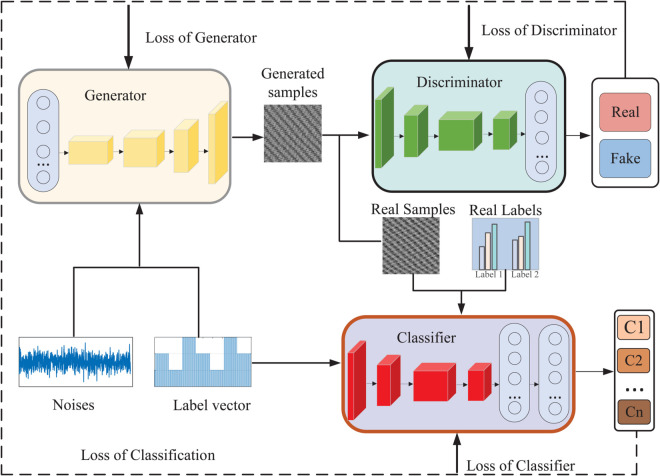
Structure of the improved ACGAN. The improved ACGAN adds a separate neural network as a classifier, improving the compatibility of discriminative accuracy and correct classification.

The traditional ACGAN adds classification labels with error information compared to GAN, which makes it possible to generate higher quality samples, and the discriminator then has the ability to discriminate and classify at the same time. However, the neural networks of the discriminator’s discrimination and classification modules share hyperparameters and weights, and if one of them is wrong, the quality of the final generated samples will be greatly affected. Therefore, To enhance the compatibility between discrimination accuracy and classification effectiveness, it is proposed to introduce a separate neural network that functions independently as a classifier. This approach would enable the discriminator to focus exclusively on assessing the authenticity of the samples, and the output of the classifier exerts an influence on the generator to classify the results, enabling the generator to generate error samples of multiple modes. The structure of the improved ACGAN is shown in Fig 5.

The approach of employing independent classifiers within GAN closely resembles the structure of ControlGAN. This framework comprises three neural networks: a discriminator, a generator, and an independent classifier, which incorporates a balancing parameter to reconcile the learning processes of the GAN architecture with those of the decoder structure [47]. Notably, in ControlGAN, the classification loss of the generated data is exclusively utilized to train the generator. In contrast, the classifiers within the VAE-IACGAN pro-posed in this paper are trained using both real and generated datasets, thereby mitigating the risk of overfitting to imbalanced data.

As illustrated in the figure, the enhanced ACGAN architecture comprises three independent neural networks. The generator includes a fully connected layer followed by a two-dimensional transposed convolutional layer, which transforms the input Gaussian noise into generated samples through an inverse convolution operation. The discriminator and classifier consist of a fully connected layer and a two-dimensional convolutional layer, where the generated samples are downscaled, judged and categorized separately by convolutional operations. ReLU is chosen as the activation function of each convolutional layer, and Leaky ReLU is the activation function of each transposed convolutional layer. To accelerate convergence, enhance the model’s generalization capabilities, and accommodate higher learning rates, Batch Normalization (BN) is incorporated into the convolutional and deconvolutional layers of both the generator and the classifier. For the discriminators, BN cannot be used as it may break its Lipschitz continuity condition. Lipschitz continuity is an important property of GAN stability that ensures smoothness of the gradient and thus helps stabilize the training process. The operations of “divide variance” and “multiply scale factor” in BN may cause the discontinuity of gradient, which affects the stability of GAN training. Meanwhile, the optimizer of the three neural networks selects the better adaptive moment estimation (Adam) by comparing Adam, SGD, RMSprop and so on.

### Improvement of ACGAN in combination with VAE

In the domain of fault diagnosis, the availability of a sufficient number of fault samples is a prerequisite for effectively training the model, Nevertheless, obtaining an adequate number of fault samples in real industrial settings poses significant challenges. Both VAE and ACGAN are commonly used generative models in the deep learning field, and they both enable data augmentation. However, both have certain shortcomings when applied individually. The VAE model obtains the distribution of the original data through resampling, but generally generates samples of low quality; the ACGAN model generates samples with diversity, but the training process is unstable, i.e., it is difficult to maintain the dynamic equilibrium between the generator and the discriminator, and the discriminator simultaneously performs the tasks of discrimination and classification, which may lead to gradient conflicts that adversely impact the quality of the generated samples. Therefore, the VAE-IACGAN (VAE-Improved ACGAN) model is proposed by combining the data generation of the VAE model and the adversarial learning mechanism of the ACGAN model.

**Fig 6:**
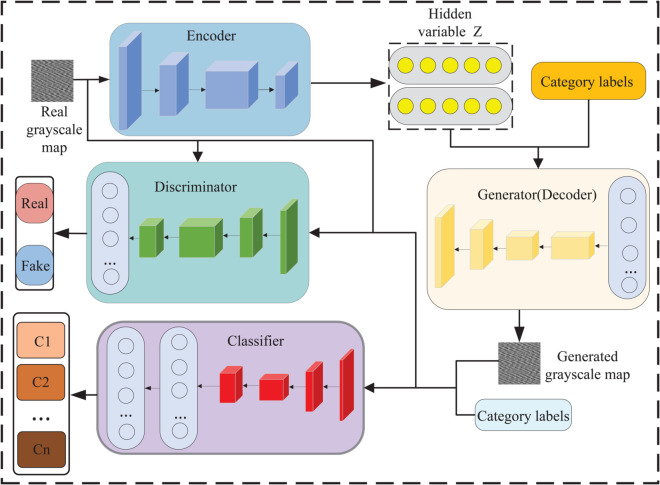
Schematic diagram of the VAE-IACGAN structure. Combining the probabilistic generation capability of VAE with the conditional control mechanism of the improved ACGAN, high-quality data generation with specified class attributes is realized after joint training.

The model is comprehensively delineated into four distinct components, which are encoder, decoder (generator), discriminator, and classifier [48,49]. That is, the process of encoding real data is added to the improved ACGAN mentioned above, and the hidden variable z with prior information is utilized as the input to the generator instead of the original random Gaussian noise, making the model much more expressive. The structure of the VAE-IACGAN model is shown in Fig 6.

As shown in Fig 6, the decoder of the VAE shares the same network module and parameters with the generator of IACGAN. The encoder network maps real grayscale images to latent variables z, and the generated samples are obtained by the generator network from the learned distribution. The discriminator network utilizes the structure of IACGAN to determine whether an input sample is synthetic or real. The classification network employs a SoftMax classifier to output the category labels of the samples.

The conventional encoder network is composed of several fully connected layers, aimed at enhancing the feature extraction capability of the network model, this paper introduces convolution and pooling operations on the original basis, the en-coder is composed of multiple convolutional blocks, each convolution block contains convolution layers, pooling layers and other operations for data compression and feature extraction. The loss function of the coding network consists of two parts, i.e., the KL scatter loss and the reconstruction loss. The formulas are as follows:

$$L_{VAE}=L_{KL}+L_{rec}$$ (10)

And

$$L_{KL}=-\frac{1}{2}(1+ln(\sigma^{2})-\mu^{2}-\sigma^{2})$$ (11)

$$L_{rec}=∥x-\tilde{x}{∥}_{2}$$ (12)

$$L_{VAE}=∥x-\tilde{x}{∥}_{2}-\frac{1}{2}(1+ln(\sigma^{2})-\mu^{2}-\sigma^{2})$$ (13)

Where *x* and $\tilde{x}$ represent the input signal and the reconstructed signal, μ is the mean, and $\sigma^{2}$ is the variance.

The goal of VAE is to learn an efficient representation of the input data by minimizing this total loss function while maintaining continuity and structure in the latent space, which allows VAE to generate new data points that are similar to the training data.

Decoding network i.e. generator. It includes a fully connected layer alongside a two-dimensional transposed convolutional layer, which facilitates the input of the encoded latent variables via the transposed convolution operation. This configuration aims to effectively learn the underlying data distribution, thereby generating synthetic samples that closely resemble the real data, and continuously improves the quality of the generated samples by training against the discriminator.

The discriminative network, otherwise known as the discriminator, is comprised of a fully connected layer and a two-dimensional convolutional layer. Through the convolution operation, the generated sample features are extracted, downscaled, feature compressed, and then processed through the fully connected layer. Ultimately, a probability value is produced, indicating the classification of the input data as either authentic or artificially generated.

Classification network i.e. classifier. The architecture comprises a fully connected layer in conjunction with a 2D convolutional layer. Classifiers are incorporated to enhance the compatibility of discrimination and classification tasks, thereby improving the model’s generalization capability and increasing the diversity of the generated samples.

The parameters of the proposed overall VAE-IACGAN network structure are shown in Table 2. Where the inputs to the encoder and discriminator are 64×64×1 grayscale maps.

**Table 2:** Model specific parameters of VAE-IACGAN.

Networks	Structure composition	Parameters	Activation function
Encoder	Conv2D(3×3×32)	kernel=3×3, stride=1	Leaky ReLU
	Conv2D(3×3×64)	kernel=3×3, stride=2	Leaky ReLU
	Maxpool(16×16×64)	pool_size=2×2	–
	Dense(8×8×128)	–	–
Decoder(Generator)	Conv2Dtranspose(16×16×128)	kernel=5×5, stride=2	ReLU
	Conv2Dtranspose(32×32×64)	kernel=5×5, stride=2	ReLU
	Conv2Dtranspose(64×64×32)	kernel=5×5, stride=2	Leaky ReLU
	Conv2Dtranspose(64×64×1)	kernel=3×3, stride=1	Tanh
Discriminator	Conv2D(32×32×32)	kernel=4×4, stride=2	Leaky ReLU
	Conv2D(16×16×64)	kernel=4×4, stride=2	Leaky ReLU
	Conv2D(8×8×128)	kernel=4×4, stride=2	Leaky ReLU
	Conv2D(4×4×256)	kernel=4×4, stride=2	Leaky ReLU
	Dense(1)	–	Sigmoid
Classifier	Conv2D(64×64×32)	kernel=3×3, stride=1	ReLU
	Conv2D(64×64×64)	kernel=3×3, stride=1	ReLU
	Conv2D(32×32×128)	kernel=3×3, stride=2	ReLU
	Conv2D(16×16×256)	kernel=3×3, stride=2	ReLU
	Global average pooling(1×1×256)	–	–
	Dense(256)	–	ReLU
	Dense(Number of fault categories)	–	Softmax

### Loss function based on Wasserstein distance and gradient penalty

As previously stated, the JS divergence is employed to evaluate the discrepancy between the distributions of real and generated data. However, the discontinuity of the JS divergence renders the training of extremely large and small datasets unstable, potentially leading to gradient disappearance and pattern collapse during the training process. This, in turn, may compromise the quality of the generated samples. Consequently, the Wasserstein distance is utilized in lieu of the JS scatter to define the loss function of the ACGAN.

In ACGAN, the discriminator is required to adhere to the Lipschitz continuity constraint to ensure that it exhibits continuous smoothness within the input space, thereby contributing to the stability and convergence of ACGAN training. To satisfy this condition, the weight parameters of the discriminator are typically constrained to a range through weight cropping. Nevertheless, weight cropping may result in the weight parameters converging towards the boundaries of the cropping process during the training phase. This may impede the model’s capacity to learn effectively, as the weight updates may cease to alter after reaching the boundaries. Additionally, weight cropping may influence the dynamic nature of the model training process. In certain instances, this could potentially lead to an unstable training process or necessitate a greater number of iterations to achieve convergence.

To tackle the previously mentioned issue, a novel loss function, based on the Wasserstein distance, is introduced, along with a gradient penalty term, which is designed to satisfy the Lipschitz continuity condition [50]. The gradient penalty term can be expressed as follows:

$$L_{gp}=\lambda E_{\bar{x}∼P_{\bar{x}}}[(∥\nabla_{\bar{x}}D(\bar{x}){∥}_{2}-1{)}^{2}]$$ (14)

where the variable $∥\;\cdot \;{∥}_{2}$ denotes the L2 Norm, ∇ denotes the gradient operator, *λ* signifies the penalty term coefficient. $\bar{x}=\varepsilon x\;+\;(1\;-\;\varepsilon )\tilde{x}$, where $x∼P_{data}$ means that *x* is drawn from the original data distribution *p*_*data*_ and $\tilde{x}∼P_{g}$ means that $\tilde{x}$ comes from the generative data distribution *P*_*g*_ where “*ε*” is the uniform distribution of [0, 1]. $P_{\bar{x}}$ is the sampling distribution that represents the gradient penalty term, which ranges from the intermediate distribution between the generated data distribution and the true data distribution.

In this paper, We propose enhancing the loss function of the fundamental ACGAN by incorporating Wasserstein distance in place of JS divergence, and the new loss function is as follows:

$$L_{C}^{R}=E_{x∼P_{r(x)}}[-logP(c=c_{r}|x)]$$ (15)

$$L_{C}^{G}=E_{z∼P_{z}}\left[-\log P\left(c=c_{g}|G\left(z,c_{g}\right)\right)\right]$$ (16)

$$L_{D}=E_{x∼P_{r(x)}}\left[D(x)\right]-E_{z∼P_{z}}\left[D(G(z,c_{g}))\right]+L_{gp}$$ (17)

$$L_{G}=-E_{z∼P_{z}}\left[D(G(z,c_{g}))\right]+0.5\times L_{C}^{R}+0.5\times L_{C}^{G}L_{C}=\delta_{1}L_{C}^{R}+\delta_{2}L_{C}^{G}$$ (18)

where *L*_*D*_, *L*_*C*_, and *L*_*G*_ are the loss functions of discriminator D, classifier C, and generator G, respectively, where *L*_*gp*_ is the gradient penalty term added to make discriminator D satisfy Lipschitz continuity. $L_{C}^{R}$ and $L_{C}^{G}$ are the loss functions of the classifier for real and generated samples. $\delta_{1}$ and $\delta_{2}$ are the scaling factors for the classification loss.

In general, a 1-Lipschitz function is defined as a function whose gradient is not greater than 1. In Eq (17), the penalty term is designed to discourage gradient paradigms that deviate from 1, thereby ensuring that all gradient paradigms converge to 1. This approach has been empirically shown to result in faster convergence and enhanced optimization out-comes. This is because, when the discriminator is optimized, it essentially becomes a gradient paradigm within the distributions *P*_*g*_ and *P*_*r*_.

### Training in the construction of diagnostic models

**Fig 7:**
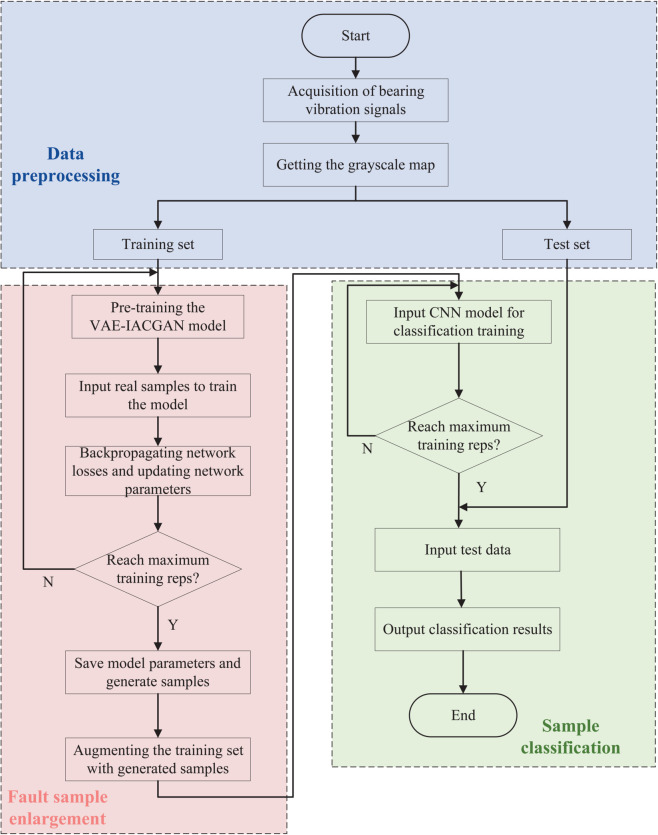
Bearing Fault Diagnosis Flowchart. The blue part is the data preprocessing part, the pink part is the fault sample generation part, and the green part is the fault classification part.

The comprehensive framework of the fault diagnosis model encompasses three principal components: data preprocessing, fault sample augmentation, and sample classification. The training process is illustrated in Fig 7.

Step 1 is data preprocessing: the original data set for high-speed train wheelset bearings is typically one-dimensional time-domain vibration signals, which present several challenges. These include dense sampling points, inconspicuous fault characteristics, and redundant information. To address these issues, the one-dimensional time-domain vibration signal is transformed into a two-dimensional gray-scale representation. Additionally, the preprocessed dataset is partitioned into a training set and a test set.

Step 2 is the expansion of fault samples: the grayscale map of the training set corresponding to various types of fault samples is input to the VAE-IACGAN model for training. Once the maximum number of training times has been reached, the model parameters are saved, and the generated samples of various types of fault samples are obtained. The original samples are then mixed with the generated samples, thereby expanding the data samples.

Step 3 is sample classification: the data-enhanced training set is input to the convolutional neural network (CNN) for classification model training. The trained CNN is then used to classify faults with the test set. This process utilizes the powerful feature extraction capability of CNN to accurately classify various types of fault samples.

## Experimental validation

In order to verify the effectiveness of the proposed method, this chapter conducts a data enhancement and intelligent diagnosis study for train wheelset bearings. In the study. The programming language used is Python 3.8, and the deep learning framework is PyTorch 1.12. All experiments are performed on a computer with an Intel Core i7-12700K CPU @ 3.60 GHz, 32 GB RAM, and a NVIDIA GeForce RTX 3080 GPU (with 10 GB of memory), running a Windows 10 64-bit operating system.

**Fig 8:**
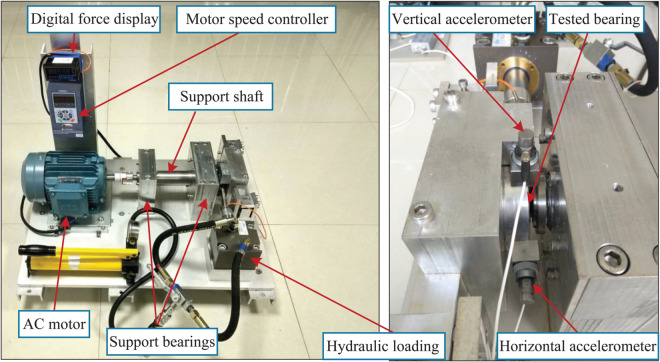
Rolling Bearing Test Stand. Tested bearing as the test object, can replace different types of bearings, AC motor drive bearing according to the set speed operation, Hydraulic loading simulation of the actual working conditions of the complex load, Digital force display real-time display of the loading force value, to ensure the accuracy of the load.

The inspection data of the wheelset bearing of a high-speed train under actual working conditions is nonlinear and lacks smoothness. Additionally, there is a paucity of available fault data, and it is challenging to simulate the fault data artificially. As a result, traditional methods are unable to meet the diagnostic accuracy requirements. In light of these challenges, a VAE-IACGAN-CNN model has been proposed for the fault diagnosis task. To assess the efficacy of the model for fault diagnosis, the methodology was validated using the Xi’an Jiaotong University (XJTU) bearing dataset [51]. The test apparatus is depicted in Fig 8.

The XJTU-SY bearing dataset is acquired during the operational period from normal functioning to failure, characterizing the degradation features of bearings throughout their operational lifespan. Two accelerometers were mounted on the vertical and horizontal directions of the tested bearing housing respectively. Since the load was applied along the horizontal direction, the vibration signals from this orientation contain more failure-related information of the bearing. Therefore, the horizontal vibration signals were selected to validate the methodology proposed in this study.

The test bearing employed was an LDK UER204 rolling bearing, whose relevant parameters and accelerated life test conditions are systematically documented in Table 3.

**Table 3:** LDK UER204 rolling bearing parameters.

Parameter Name	Value	Parameter Name	Value
Inner race diameter/mm	29.30	Ball diameter/mm	7.92
Outer race diameter/mm	39.80	Number of balls	8
Bearing mean diameter/mm	34.55	Contact angle/(°)	0
Load rating (dynamic)/N	12820	Load rating (static)/N	66500

The dataset encompasses three distinct operating conditions with varying loads and rotational speeds. Each condition was tested using five bearings. The sampling frequency was set to 25.6 kHz, with each sample containing 32,768 data points corresponding to a 1.28 second acquisition duration. Sampling intervals were maintained at 1 minute. These experimental configurations across three operating conditions yielded 15 test results, which included corresponding operational parameters, total data sample quantities, actual lifespans, and failure locations. For experimental validation, data representing four different health states were selected, with detailed specifications of these four data categories provided in Table 4.

**Table 4:** Bearing dataset for four faults.

Fault number	Operation condition	Fault element	Bearing lifetime	Sampling point
Fault1	37.5Hz/11kN	Inner race	8h11min	491
Fault2	40Hz/10kN	Ball	12h26min	2496
Fault3	37.5Hz/11kN	Outer race	5h39min	339
Fault4	40Hz/10kN	Inner race	6h11min	371

**Fig 9:**
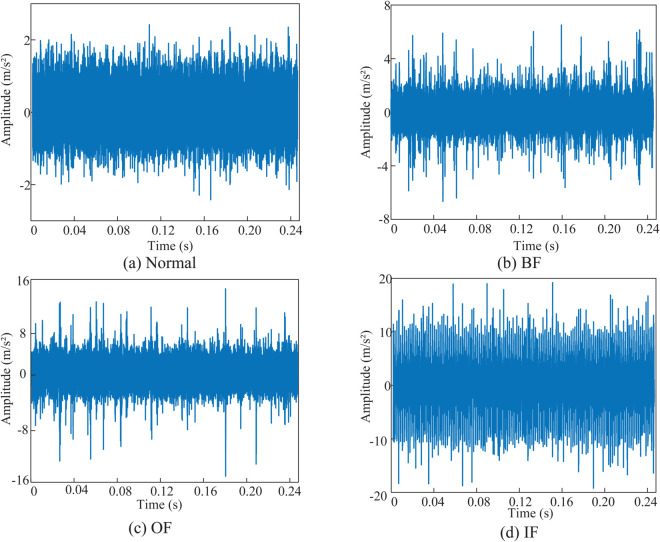
Time domain waveforms for different health states. (a) Waveforms in the time domain of the healthy state of the bearing. (b) Waveforms in the time domain of the rolling element failure of the bearing. (c) Waveforms in the time domain of the outer ring failure of the bearing. (d) Waveforms in the time domain of the inner ring failure of the bearing.

Therefore, fault data corresponding to the operating conditions listed in the table were selected for experimental validation. Fig 9 presents the time-domain waveforms of horizontal vibration signals under different bearing fault conditions.

The graphical results demonstrate discernible variations in vibration intensity across the four health states. Specifically, the rolling element fault manifests intermittent impact pulses in its waveform. The outer race fault exhibits pronounced modulation phenomena with significantly enhanced impact pulse density and amplitude. The inner race fault displays severe waveform distortion accompanied by disrupted periodicity, presenting dense impact pulse clusters. However, although time-domain data as one-dimensional time series can be directly fed into neural networks, this approach risks neglecting critical frequency-domain characteristics. Moreover, inherent noise contamination and amplitude variations in temporal signals may impede effective feature extraction, particularly for incipient fault detection. The non-stationarity of time-domain data, such as rotational speed fluctuations, further compromises model performance. To address these limitations, a signal-to-image transformation methodology is proposed to enhance fault feature extraction through multidimensional representation.

### Data set pre-processing

**Fig 10:**
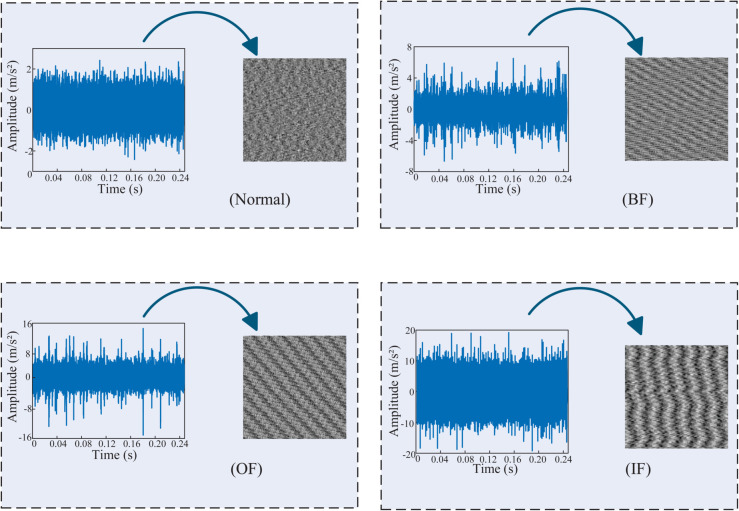
Time-domain waveforms of the bearing vibration signals and the corresponding grayscale images obtained by the transformation. (a) the waveforms of the time domain of the healthy state of the bearing are converted to grayscale. (b) the waveforms of the time domain of the rolling element failure of the bearing are converted to grayscale. (c) the waveforms of the time domain of the failure of the outer ring of the bearing are converted to grayscale. (d) the waveforms of the time domain of the failure of the inner ring of the bearing are converted to grayscale.

In this paper, a method for signal-image conversion is employed to transform the original one-dimensional vibration signals into two-dimensional gray-scale representations, which is capable of multidimensional feature extraction, i.e., the traditional one-dimensional convolution kernel can only extract features in a single dimension of time or frequency. By transforming the signal into a two-dimensional image, it is possible to extract features in both the time and frequency dimensions using a two-dimensional convolution kernel, which facilitates the capture of more complex signal patterns. The method of preprocessing is defined as follows:

$$P(j,k)=\text{round}\{\frac{L((j-1)\times M+k)-min(L)}{max(L)-min(L)}*255\}$$ (19)

where *P*(*j*,*k*) is the value of the (*j*,*k*)th element of the 2D gray-scale image matrix obtained after the transformation. Here, M denotes the dimensional extent of the grayscale image, while *L* represents an arbitrary length derived from the one-dimensional vibration signal, corresponding to a continuous array of M×M sampling points. By employing the round(·) function, each pixel value is normalized using minmax normalization, resulting in integer values ranging from 0 to 255. In this context, *L* is specified as 4096 and *M* is set to 64. The comprehensive single-image outcomes for a single sample under various conditions are illustrated in Fig 10.

It is evident that the transformed signal exhibits more pronounced features distinguishing between various health states compared to the original time-domain signal.

### Selection of hyperparameters

The number of iterations is a critical factor in the training of models; an excessively high setting may lead to increased consumption of computational resources, while a setting that is too low may result in the model’s inability to capture the complex patterns inherent in the data. To identify the optimal number of epochs, this study conducts experiments utilizing the XJTU bearing dataset. So the experiment was set up: the VAE-IACGAN model was trained using a set of datasets from the XJTU bearing dataset, and the FID (Fréchet Inception Distance) values of the real fault samples and the generated fault samples were calculated for different iterations, and the iteration Epochs with the lowest FID values were selected.

**Fig 11:**
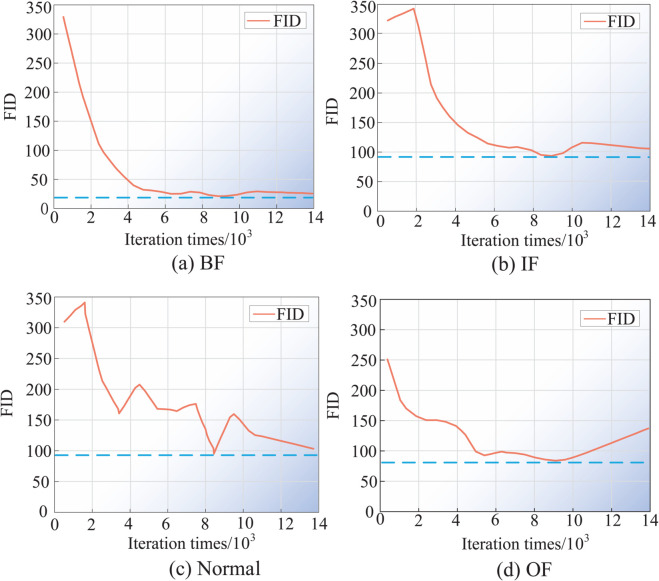
Line graph of FID values for different number of iterations. FID values for real and generated samples at different number of iterations. (a) Rolling body failure. (b) Inner ring fault. (c) Normal state. (d) Outer ring fault.

To facilitate an accurate comparison of the impact of varying iteration numbers on the generative performance of the VAE-IACGAN model, it is necessary to fix the other hyperparameters in the model and only vary the size of the iteration number. The batch size is configured to 64, with a learning rate established at 0.0001, and the lengths of the encoder output implied variable *z* and the decoder input implied variable *z* are both 512 and obey a normal distribution. Fig 11 presents the FID values comparing the real fault samples with the generated fault samples across various iteration counts.

As can be seen in Fig 11, the samples of all four modes were trained under the VAE-IACGAN model for 14,000 iterations, and from the distribution of the FID values of the four modes, the BF, IF, and OF faults reached the global minimum value of the FID value when the number of iterations reached 9,000 and 10,000 iterations, in which the BF fault had the lowest value of FID of 22.583 and the IF fault had the lowest value of FID of 92.352, and the lowest FID value for OF fault is 90.556. The FID value for normal samples reaches the global minimum at 9,000 iterations, at 102.415. Since the FID value is utilized to evaluate the divergence between the distributions of real and generated samples, the smaller the value, the more diverse and high quality the generated samples are. Therefore, it is shown in this experiment that the number of iterations is 9,000 or 10,000, the distribution distance between real and generated samples is the closest, and the generated samples have the most diverse dataset and the highest quality. Taking into consideration, the VAE-IACGAN model in this paper selects the number of iterations Epochs = $9\;\times \;10^{3}$ as one of the hyperparameters of the model.

**Fig 12:**
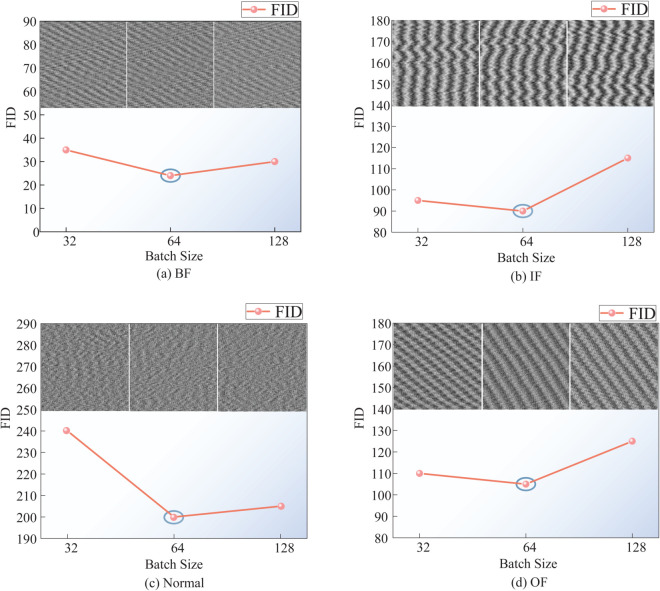
The FID values point-line plot for different batch sizes. FID values for real and generated samples at different batch sizes. (a) Rolling body failure. (b) Inner ring fault. (c) Normal state. (d) Outer ring fault.

Building upon the first experimental configuration, the VAE-IACGAN framework was fixed at $9\;\times \;10^{3}$ iterations while maintaining identical hyperparameter settings. Three batch sizes (32, 64, 128) were systematically evaluated by computing FID values between authentic and synthesized fault samples. The optimal batch size was determined through minimum-FID selection criteria. Fig 12 comparatively illustrates FID metrics across different health states and batch sizes, quantifying the distributional discrepancies between genuine and generated fault samples under varying experimental configurations.

Fig 12 demonstrates grayscale images of generated samples across varying health states under different batch sizes (32, 64, 128). Visual inspection reveals indistinguishable differences between generated samples, necessitating the employment of FID metrics to quantitatively evaluate both diversity and perceptual quality of the generated fault data.

Experimental results in Fig 12 demonstrate that at a batch size of 64, the FID values for rolling element faults (22.04), inner race faults (89.12), outer race faults (198.43), and normal samples (108.68) simultaneously reached their minima. To balance generative diversity and fidelity, the batch size of 64 was consequently selected as the optimal hyperparameter configuration for the VAE-IACGAN framework.

In the hyperparameter selection experiment, the Fréchet Inception Distance (FID) is relatively higher than that in the field of natural images. Pan et al. [35] pointed out that due to the nonlinear and non-stationary characteristics of vibration signals, the FID in small-sample scenarios is generally higher than that in the conventional image field. This is attributed to the small-sample and data imbalance issues in mechanical fault diagnosis; furthermore, the grayscale images studied in this paper only reflect the amplitude variation of vibration signals, resulting in a single feature dimension. In addition, key fault features such as impact pulses are sparsely distributed in the pixel space, which leads to a relatively high calculated FID value between generated samples and real samples. Moreover, the complexity of vibration signals varies significantly among different fault types: For ball fault (BF), the impact features are regular and concentrated, leading to low generation difficulty and thus the lowest FID value of 22.583. For inner race fault (IF) and outer race fault (OF), affected by wheel-rail impact and load fluctuation, the signals contain multi-frequency modulation components, which increases the difficulty of capturing fault features; consequently, their FID values reach 92.352 and 90.556, respectively. Normal samples have no clear fault features, resulting in stronger signal randomness and the highest FID value of 102.415. This difference is consistent with the complexity distribution law of fault signals in practical engineering.

### Changes in losses during training

**Fig 13:**
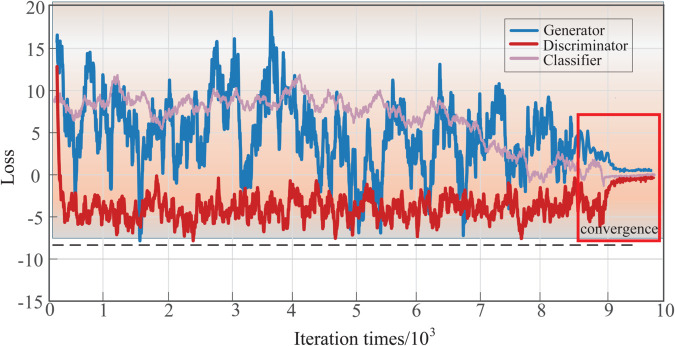
Loss function variation curve. The blue part is the generator loss function variation curve, the red is the discriminator loss function variation curve, and the pink is the classifier loss function variation curve.

To visualize the model training process, Fig 13 illustrates the evolution of the loss function in relation to the number of iterations throughout the training process.

As illustrated in Fig 13, the classifier loss values display negligible variations during the early stages of training, with the loss swiftly converging toward zero following 7,000 iterations. During the initial phase of training, the loss values of both the discriminator and generator demonstrate significant fluctuations. However, as the number of iterations increases, these fluctuations tend to stabilize, eventually reaching a Nash equilibrium after approximately $9\;\times \;10^{3}$ iterations. This indicates that the adversarial training process has been sufficiently optimized to generate high-quality samples.

### Quality assessment of the generated samples

**Fig 14:**
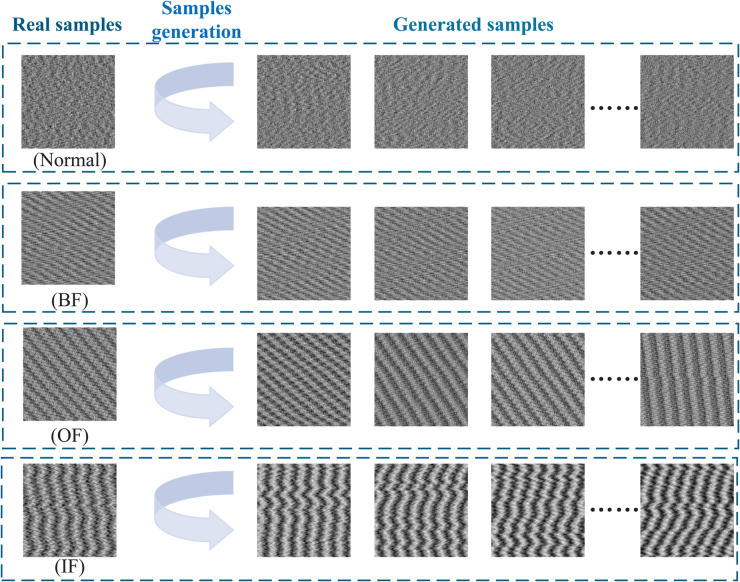
Schematic diagram for generating bearing samples. The left side is the real sample and the right side is the corresponding generated sample.

In this paper, the VAE-IACGAN model generates four types of faults in wheel-set bearings for high-speed trains: outer ring faults, inner ring faults, rolling element faults, and cage faults. Real samples are used to train the model to generate samples for expanding the dataset. The generated samples are then analyzed and evaluated for quality.The schematic of sample generation is shown in Fig 14.

To evaluate the effectiveness of the model in sample generation, comparison experiments have been conducted. The identical real sample dataset has been utilized to train both the ACGAN and the VAE-GAN, with the corresponding samples generated under the respective models. Consequently, three metrics have been employed to assess the quality of the generated samples at the data level: the structural similarity index (SSIM), the maximum mean difference (MMD), and the peak signal-to-noise ratio (PSNR).

#### Quality assessment of structural similarity indicators.

The Structural Similarity Index (SSIM) is designed to evaluate the similarity between two images by considering three key factors: brightness, contrast, and structural integrity, mathematically defined as follows:

$$SSIM(x,y)=l(x,y)\;\cdot \;c(x,y)\;\cdot \;s(x,y)\;=\left(\frac{2\mu_{x}\mu_{y}+C_{1}}{\mu_{x}^{2}+\mu_{y}^{2}+C_{1}}\right)\cdot \left(\frac{2\sigma_{x}\sigma_{y}+C_{2}}{\sigma_{x}^{2}+\sigma_{y}^{2}+C_{2}}\right)\cdot \left(\frac{\sigma_{xy}+C_{3}}{\sigma_{x}\sigma_{y}+C_{3}}\right)$$ (20)

where *x* and *y* represent two grayscale images, while $\mu_{x}$, $\sigma_{x}$, $\mu_{y}$ and $\sigma_{y}$ denote the means and standard deviations of *x* and *y*, respectively. The term $\sigma_{xy}$ represents the covariance between *x* and *y*. The constants $C_{1}=\;{\left(0.01V\right)}^{2}$, $C_{2}\;=\;{\left(0.03V\right)}^{2}$, and $C_{3}=\;0.5\;C_{2}$ are introduced to avoid a zero denominator, where *V* signifies the pixel value, typically ranging from 0 to 255.

A higher SSIM value signifies a greater level of similarity between the two images, which reflects an enhanced quality in the generated samples. In this study, a total of 30 generated samples and 30 samples from the real dataset were randomly selected to evaluate their SSIM quality. The five categories of bearing states are classified as normal samples, outer ring faults, inner ring faults, rolling element faults, and cage faults, designated as C0, C1, C2, C3, and C4, respectively. The mean value of the SSIM is calculated 1000 times and serves as the final evaluation value. The specific values are presented in Table 5.

**Table 5:** SSIM values of generated samples and real samples for different health states under the three models.

Model \ Label	CO	C1	C2	C3	C4
VAE-IACGAN	0.4562	0.4861	0.4286	0.4734	0.4179
ACGAN	0.2511	0.2374	0.2636	0.2971	0.1855
VAE-GAN	0.3124	0.3368	0.3477	0.3214	0.3714

As indicated in Table 5, there exists a minor difference in the SSIM values across samples corresponding to various classes of health states, and for VAE-IACGAN, the values are above 0.4. Compared to the two models ACGAN and VAE-GAN, the samples generated by the new model are of higher quality when it comes to the SSIM indicator.

**Fig 15:**
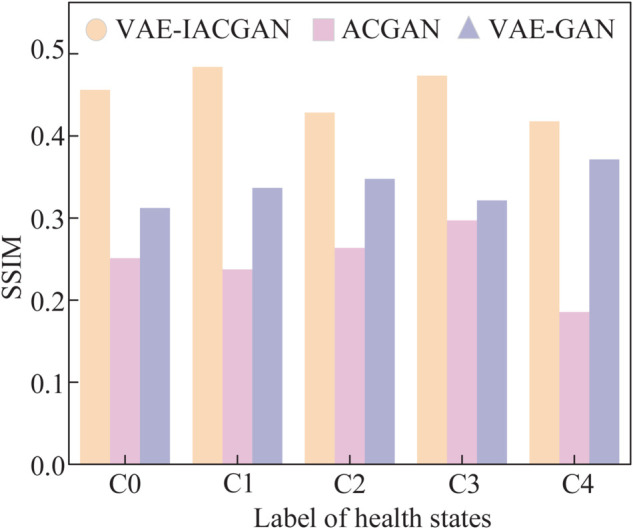
Comparison of SSIM results. Comparison of SSIM assessment results between real samples and generated samples generated by the three models.

The comparison of SSIM evaluation results for the VAE-IACGAN, ACGAN, and VAE-GAN generated samples is shown in Fig 15.

#### Maximum mean difference quality assessment.

The Maximum Mean Difference (MMD) metric is utilized to quantify the dis-parity between the distributions of two sample sets within the framework of Hilbert space, and is widely used in machine learning and statistics, especially in areas such as domain adaptation and generative adversarial networks. Therefore, this method is also employed as an evaluative metric to assess the quality of generated samples. MMD is defined as follows:

$$\text{MMD}(P,Q;ℋ)=\underset{\|f{\|}_{ℋ}\leq 1}{\sup}\left\{{𝔼}_{x∼P}[f(x)]-{𝔼}_{y∼Q}[f(y)]\right\}$$ (21)

where f(·) is a function within the Reproducing Kernel Hilbert Space (RKHS), and ℋ denotes the RKHS itself. *P* and *Q* represent two distributions to be compared, corresponding to the real samples and the generated samples, respectively. The notation $|f{|}_{ℋ}\leq 1$ indicates the RKHS norm of the function f(·), which is constrained to be within 1.

**Fig 16:**
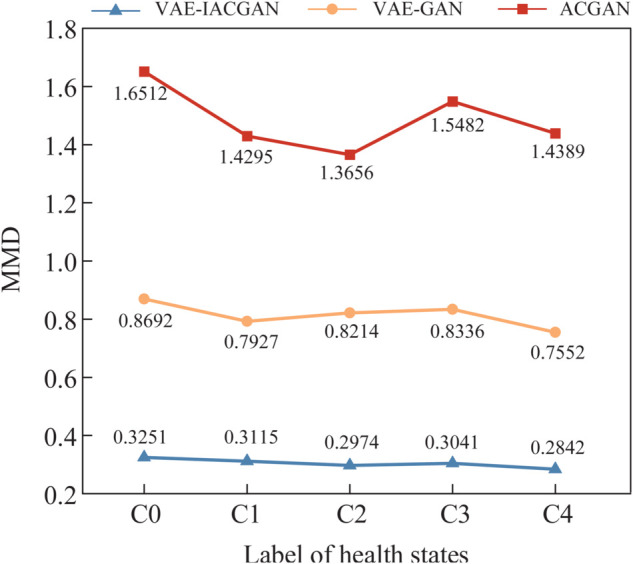
Comparison of MMD results. Comparison of MMD assessment results between real samples and generated samples produced by the three models.

In contrast to SSIM, MMD is a measure of the difference between two distributions; the smaller the difference, the smaller the MMD value, indicating the higher quality of the generated sample. Again, 30 samples from each of the above experiments were taken to calculate the MMD value 1000 times and then the average value was obtained as the final assessment value, and a comparison of the quality assessment results is shown in Fig 16.

As depicted in Fig 16, the MMD values for the VAE-IACGAN model are significantly lower than those for both the VAE-GAN and ACGAN models. This observation indicates that, at this level of analysis, the samples generated by the VAE-IACGAN model more closely approximate the characteristics of the real samples.

#### Peak signal-to-noise ratio quality assessment.

The Peak Signal to Noise Ratio (PSNR), a quantitative metric for assessing signal quality, represents the ratio of the maximum signal power to the power of the noise, typically expressed in decibels (dB). PSNR is derived from the Mean Squared Error (MSE), which quantifies the difference between an original image *I* of dimensions m×n and a noisy image *K* subjected to the introduction of noise, defined as follows:

$$MSE=\frac{1}{mn}\sum_{i=0}^{m-1}\sum_{j=0}^{n-1}[I(i,j)-K(i,j){]}^{2}$$ (22)

where *I*(*i*,*j*) refers to the pixel values of the original image, whereas *K*(*i*,*j*) signifies the pixel values of the generated image.

Then PSNR is defined as:

$$PSNR=10\cdot \log_{10}\left(\frac{MAX_{I}^{2}}{MSE}\right)=20\cdot \log_{10}\left(\frac{MAX_{I}}{\sqrt{MSE}}\right)$$ (23)

where *MAX*_*I*_ represents the maximum pixel value of the image and *MSE* denotes the mean square value of the difference between the signal and the original signal.

As with SSIM, a higher PSNR value indicates a higher quality image. In general, a value exceeding 40 dB indicates a very close approximation of the original image, a PSNR value ranging from 30 to 40 dB suggests that the overall quality of the image is considered good, whereas a value below 30 dB indicates that the image quality is suboptimal.

**Fig 17:**
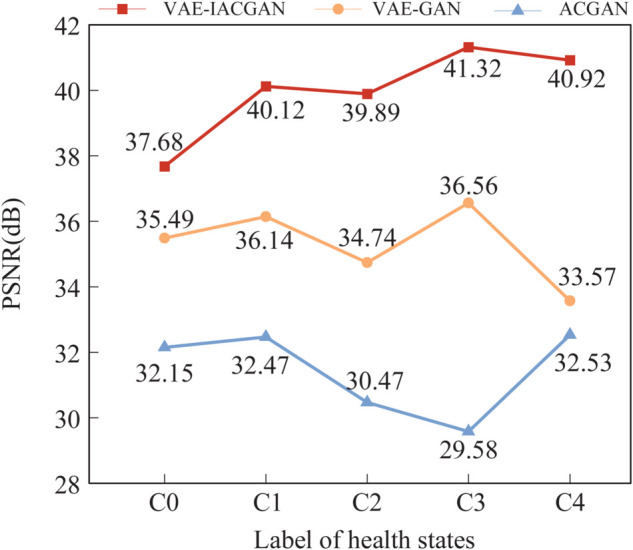
Comparison of PSNR results. Comparison of PSNR assessment results between real samples and generated samples produced by the three models.

The PSNR evaluation was carried out with the same samples as above, and the average of the 1000 calculated PSNRs was taken as the final evaluation value, and the results of the PSNR values of the generated samples with the other two models are plotted as shown in Fig 17.

The results presented in Figs 15, 16, and 17 demonstrate that the SSIM and PSNR values for the samples generated by the VAE-IACGAN are consistently higher than those for the VAE-GAN and ACGAN models. Additionally, the MMD values for the VAE-IACGAN are consistently lower than those of the other two models. This evidence suggests that, at the data level, the samples generated by the VAE-IACGAN exhibit superior quality and their distribution is more closely aligned with that of the real samples.

#### T-SNE feature level quality assessment.

Beyond the assessment performed at the data level, in order to visualize the feature distribution of the generated samples with respect to the real samples, the T-SNE (T-Distributed Stochastic Neighborhood Embedding) technique was used. Mapping the high-dimensional data into a low-dimensional space allows similar data points to be closer together in the new space, while dissimilar data points are separated, thus revealing the underlying structure of the data.

**Fig 18:**
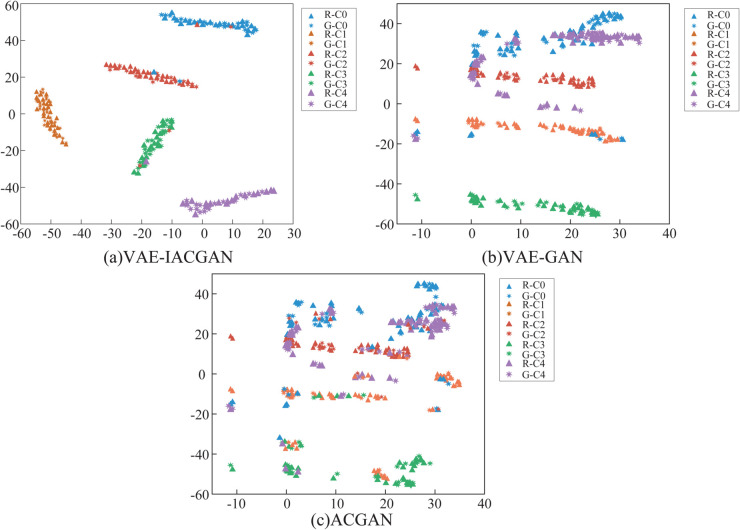
Feature visualization results. T-SNE visualization results of samples with different health states generated by the three models versus real samples with different health states. (a) VAE-IACGAN. (b) VAE-GAN. (c) ACGAN.

The relevant features are extracted from the generated and real samples and visualized using the T-SNE method, and the visualization results of the three models are shown in Fig 18.

As illustrated in Fig 18(a), the 2D feature distributions of the samples generated by the VAE-IACGAN demonstrate a significant degree of clustering alongside the real samples. As shown by Fig 18(b),the generated samples corresponding to the C1, C2, and C4 health states in the VAE-GAN exhibit a pronounced clustering within the 2D feature distributions of the real samples, whereas the distributions of the remaining health states appear to be more intermixed. As depicted in Fig 18(c), the visualization result of ACGAN is chaotic and the clustering effect is not good. This indicates that the samples generated by VAE-IACGAN at the feature level have higher similarity with the real samples.

### Fault diagnosis based on CNN with expanded samples

#### Feasibility of fault diagnosis with small samples.

In this section, the samples generated by the VAE-IACGAN are utilized in con-junction with a modest set of real samples to facilitate the training of the CNN mod-el, and the network configuration is the same as that of the classifiers in section Improvement of ACGAN in combination with VAE. To assess the fault diagnosis performance of the VAE-IACGAN-CNN in scenarios characterized by limited sample sizes, and to simulate the rich data under multiple operating conditions that are sometimes difficult to be collected in real industrial environments, only the grayscale maps of the bearing signals under one operating condition are selected as the training samples. Four different small sample training sets A, B, C, D and test set T were constructed from real samples and generated samples. Ten experiments were conducted under identical conditions, and the aver-age of these trials was computed as the final outcome. The diagnostic results across various datasets are presented in Table 6.

**Table 6:** Fault diagnosis results of wheelset bearings of high-speed trains under small samples.

Datasets	Fault type and sample size					Proportion of samples	Accuracy
	Normal	OF	IF	BF	CF	(Real: Generation)	(%)
A	20	20	20	20	20	1:0	68.82±1.26
B	40	40	40	40	40	1:1	72.54±0.89
C	60	60	60	60	60	1:2	86.25± 0.67
D	80	80	80	80	80	1:3	92.67± 0.58
E	100	100	100	100	100	1:4	95.48± 0.42
T	100	100	100	100	100	1:4	93.25± 0.47

As shown in Table 6, under Dataset A, the average fault diagnosis accuracy achieved by using only real small-sample grayscale images is merely 68.82%. After the sample dataset is augmented by VAE-IACGAN, the accuracy is improved to varying degrees. Specifically, under Dataset E—where the ratio of real samples to generated samples reaches 1:4—the accuracy can reach 95.48%. Moreover, as the proportion of generated samples increases, the standard deviation decreases to ±0.42%, indicating that the inclusion of generated samples effectively reduces the model’s sensitivity to the randomness of the original small samples. Experimental results demonstrate that the VAE-IACGAN-CNN model can address the issue of low fault diagnosis accuracy under small-sample scenarios by augmenting the dataset with generated samples.

#### Hyperparametric sensitivity testing.

To verify the influence of the number of iterations (Epochs) and batch size on fault diagnosis accuracy, this section adjusts the number of iterations (6000, 9000, 12000) and batch size (32, 64, 128) separately based on the hyperparameter combination with the optimal FID value (Epochs = 9000, Batch Size = 64). Fault diagnosis experiments are conducted on Dataset E (real samples: generated samples = 1:4) using the same VAE-IACGAN-CNN model. The results are shown in Table 7:

**Table 7:** Fault diagnosis results of the model under different hyperparameter combinations.

Hyperparametric combination	Average accuracy (%)
Epochs = 6000, Batch = 32	85.32
Epochs = 6000, Batch = 64	89.72
Epochs = 6000, Batch = 128	88.48
Epochs = 9000, Batch = 32	92.15
Epochs = 9000, Batch = 64	95.48
Epochs = 9000, Batch = 128	90.86
Epochs = 12000, Batch = 32	91.85
Epochs = 12000, Batch = 64	94.31
Epochs = 12000, Batch = 128	92.35

As can be seen from Table 7, when the number of iterations is too low (6000), the model fails to fully converge, leading to a significant decrease in accuracy; when the number of iterations is too high (12000), overfitting occurs, and the accuracy is slightly lower than the optimal value. When the batch size is too small (32), it results in unstable model training; when the batch size is too large (128), the generalization ability of the model decreases, thereby causing a decline in accuracy. Therefore, when the number of iterations is 9000 and the batch size is 64, the model achieves the highest average accuracy, which verifies the rationality of the hyperparameter selection.

#### Ablation experiments.

To verify the independent contributions of the core components in the VAE-IACGAN-CNN model, this section designs a control experiment by employing three models: the complete VAE-IACGAN-CNN model, the IACGAN-CNN model (with VAE removed), and the VAE-ACGAN-CNN model (with the independent classifier removed). Under Dataset B and Dataset E, the accuracy of each method, as well as the SSIM and MMD values of the generated samples, are presented in Table 8.

**Table 8:** Performance comparison results of ablation experiments.

Model	Dataset B	Dataset E	SSIM	MMD
VAE-IACGAN-CNN	72.54%	95.48%	0.456	0.30
IACGAN-CNN	61.32%	81.26%	0.297	0.58
VAE-ACGAN-CNN	63.85%	84.47%	0.349	0.49

As shown in Table 8, the VAE improved the accuracy by 11.22% and 14.22% on the two datasets, respectively; meanwhile, the independent classifier in the IACGAN increased the accuracy by 8.69% and 11.01% on the two datasets. This indicates that the latent variable encoding mechanism in VAE is the key to avoiding mode collapse, while the design of the independent classifier enhances the multimodal generation capability by decoupling the classification and discrimination tasks. Furthermore, the SSIM and MMD indicators of the generated samples demonstrate that the quality evaluation of the two control experiments is lower than that of the complete VAE-IACGAN model. This further verifies that the design of VAE and the independent classifier in ACGAN proposed in this paper can improve both the quality of generated samples and the accuracy of fault classification.

#### Comparison of effectiveness with other methods.

To further validate the benefits of the VAE-IACGAN model in fault diagnosis with limited sample sizes, three models:VAE-GAN, ACGAN, and DCGAN are selected as comparison methods. The three generative models are expanded for fault samples and combined with a CNN network for fault identification. The accuracy of the various methods under different training samples is shown in Table 9.

**Table 9:** Comparison of accuracy of different methods on each dataset.

Methods	Dataset B	Dataset C	Dataset D	Dataset E
VAE-GAN-CNN	59.84± 1.35	70.96± 1.02	78.24± 0.91	82.45± 0.73
ACGAN-CNN	53.85± 1.67	64.14± 1.28	70.59± 1.05	77.83± 0.95
DCGAN-CNN	51.48± 1.82	64.89± 1.31	68.47± 1.17	74.91± 1.08
VAE-IACGAN-CNN	**72.54± 0.89**	**86.25± 0.67**	**92.67± 0.58**	**95.48± 0.42**

From the information in the Table 9, it is evident that in scenarios characterized by a notably small number of training samples (as observed in Dataset B), the DCGAN - CNN model consistently demonstrates the lowest accuracy relative to the other models examined. Additionally, both small - sample methodologies, VAE - GAN - CNN and ACGAN - CNN, exhibit accuracy levels below 60%. In contrast, the VAE - IACGAN - CNN model proposed in this study achieves an accuracy of 72.54%, which is substantially greater than that of the other three methods assessed. In dataset C, the accuracies of several methods are greatly improved, and the VAE - IACGAN - CNN accuracy is still higher than the other three methods. In datasets D and E, the accuracy rates of the methods proposed in this paper are over 90%, which also confirms the superiority of the VAE - IACGAN - CNN fault classification effect.

**Fig 19:**
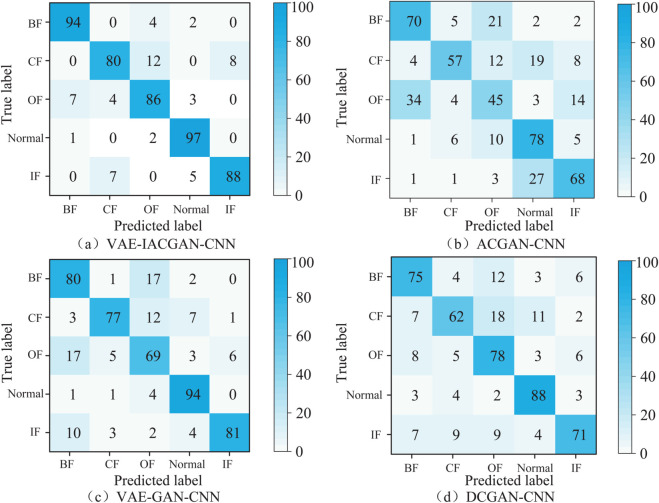
Confusion matrix of the four methods for each type of bearing fault diagnosis result on test set T. Accuracy of fault classification after assisted training of CNN with samples generated by different models. (a) VAE-IACGAN. (b) ACGAN. (c) VAE-GAN. (d) DCGAN.

To enable a comparative analysis of the four methodologies regarding their recognition capabilities for the four distinct types of bearing faults, the four models were trained on a separate basis with 40 training samples (referred to as dataset B), and the resulting diagnostic confusion matrices were plotted as shown in Fig 19. This figure depicts the performance of the four methods on a total of 100 test samples for each of the four types of faults.

As illustrated in Fig 19, when confronted with limited training data, the three comparison methods exhibit a considerable number of misclassification instances, resulting in a suboptimal diagnostic efficacy. In contrast, the VAE-IACGAN-CNN approach demonstrates a markedly superior performance in this regard. In particular, the three comparison methods are deficient in their ability to recognize inner ring faults and outer ring faults. In contrast, the method proposed in this paper demonstrates consistent and high accuracy in recognizing these two types of faults, with an overall diagnostic accuracy exceeding 80% for each type of bearing fault. The preceding analysis demonstrates that the three comparison methods are ineffective in identifying bearing faults when the training samples are limited in number. In contrast, the proposed VAE- IACGAN-CNN is capable of significantly enhancing the efficacy of fault diagnosis and facilitating the diagnosis of bearings in small samples.

## Conclusion

To overcome the challenges related to the difficulty of acquiring high-speed train wheelset bearing fault data in real industrial contexts, which subsequently results in diminished accuracy in fault identification, this paper presents a VAE-IACGAN model that effectively generates a sufficient quantity of fault samples to aid CNN in fault detection tasks. In conclusion, the methodology put forward herein is distinguished by the following advantages:

(1) The original ACGAN network is augmented with an additional neural network,which serves as a classifier. This integration enhances the compatibility between classification and discrimination. Furthermore, the output of the classifier influences the generator, enabling it to classify the results and thereby facilitating the generation of fault data across multiple modes. The VAE is introduced as the generator of the model, and the actual coding process of the VAE is used as the input of the model in place of the original Gaussian noise. This approach yields a model with enhanced expressiveness, facilitating the generation of samples that more closely align with the actual data distribution.

(2) The Wasserstein distance is adopted instead of the JS divergence to define a new loss function, which can effectively avoid the occurrence of gradient vanishing and mode collapse. In addition, a gradient penalty term is added to the loss function of the discriminator to satisfy the Lipschitz constraint, thereby ensuring the discriminator is continuous and smooth in the input space and significantly improving the stability of training. Verification via the loss function variation curve shows that after approximately 9×10^3^ iterations, the adversarial training process of the model reaches a Nash equilibrium state, without the situation where the generator and discriminator fail to converge simultaneously.

(3) Experimental verification is conducted on the Xi’an Jiaotong University (XJTU) Bearing Dataset. Firstly, a comparative analysis and quality evaluation of the samples generated by the VAE-IACGAN model, ACGAN model, and VAE-GAN model are performed from two dimensions: quantity and feature. The results indicate that the evaluation indicators of the fault samples generated by VAE-IACGAN at the data dimension are superior to those of other comparison models. Specifically, the average SSIM value is greater than 0.4, which is more than 35% higher than the optimal result among the comparison methods; the average MMD value is maintained at around 0.3, with a reduction of more than 62.5% compared to the VAE-GAN model; the PSNR value is greater than 40 dB, indicating that the generated fault samples are highly close to real fault samples. Similarly, in the evaluation indicators at the feature dimension, a comparison of the t-SNE plots of the three models reveals that the 2D feature distributions of real samples and generated samples of the proposed method are basically clustered together, while the other comparison models all exhibit a certain degree of aliasing. This demonstrates that the quality of fault samples generated by VAE-IACGAN is superior to that of other comparison models at the feature dimension. Therefore, it is verified that the samples generated by the method proposed in this paper have higher similarity to real samples.

(4) After selecting hyperparameters based on the FID value, sensitivity analysis was further conducted to verify their impact on diagnosis accuracy. Experimental results show that the optimalg244 hyperparameter combination (Epochs = 9000, Batch Size = 64) not only ensures the quality of generated samples but also enables the VAE-IACGAN-CNN model to achieve a fault diagnosis accuracy of 95.48% on Dataset E, which significantly outperforms other parameter combinations.

(5) The samples generated by the VAE-IACGAN model, combined with a small number of real samples, were used to assist in training the CNN model for fault diagnosis. Experimental verification was carried out on four constructed small-sample datasets with different settings. The results indicate that when the ratio of real samples to generated samples is 1:4, the diagnosis accuracy can reach 95.48%, representing a maximum increase of 17.65% compared with the accuracy of comparison models. This verifies that the VAE-IACGAN-CNN model can address the issue of low fault diagnosis accuracy under small-sample conditions by expanding the dataset with generated samples. Moreover, in scenarios with fewer training samples (e.g., on Dataset B), the average accuracy of the three comparison methods is all below 60%, making it difficult to effectively identify bearing faults; in contrast, the accuracy of the proposed VAE-IACGAN-CNN can still be maintained above 72%. Therefore, the VAE-IACGAN fault diagnosis method proposed in this paper achieves higher accuracy on fault datasets with different ratios, and can meet the accuracy requirements for diagnosis under conditions of scarce fault samples and unbalanced samples.

However, there exist scenarios with zero real samples in actual industrial environments. Since the method proposed in this paper requires a small amount of real samples for auxiliary training, future research directions will focus on how to generate high-quality fault samples under zero-sample conditions, establish effective fault diagnosis models, and better apply transfer learning and meta-learning.
